# Implementation Fidelity of the Transitional Care Model: A Mixed Methods Study

**DOI:** 10.1007/s43477-026-00234-5

**Published:** 2026-05-09

**Authors:** Karen B. Hirschman, Mark Toles, Christina Reilly Whitehouse, Molly McHugh, Claire Regan, Monica L. Ahrens, Alexandra L. Hanlon, Onome H. Osokpo, Kathleen McCauley, Elizabeth C. Shaid, Mark V. Pauly, Mary D. Naylor

**Affiliations:** 1 NewCourtland Center for Transitions and Health, School of Nursing, University of Pennsylvania, PA Philadelphia, United States; 2 Lenoard Davis Institute of Health Economics, University of Pennsylvania, PA Philadelphia, United States; 3 School of Nursing, University of North Carolina at Chapel Hill, NC Chapel Hill, United States; 4 M. Louise Fitzpatrick College of Nursing, Villanova University, PA Radnor, United States; 5 Center for Biostatistics and Health Data Science, College of Science, Department of Statistics, Virginia Tech, VA Roanoke, United States; 6 College of Nursing, Population Health Nursing Science, University of Illinois at Chicago, IL Chicago, United States; 7 Wharton School, University of Pennsylvania, PA Philadelphia, United States

**Keywords:** Transitional care, Randomized controlled trial, Implementation evaluation, Care transitions, RE-AIM, Fidelity

## Abstract

This study, conducted in parallel with the Multisite Replication of the Transitional Care Model, evaluated implementation of the Transitional Care Model protocol delivery (primary aim) and the randomized clinical trial protocol (secondary aim) across nine hospitals and community partners within four healthcare systems. Guided by the Practical, Robust, Implementation, and Sustainability Model and applying a concurrent mixed-methods design, outcomes were assessed using an embedded Reach, Adoption, Implementation, and Maintenance framework, excluding effectiveness. Fidelity to implementation was measured as a standardized count of protocol components delivered as intended (range 0–38). A mean fidelity score of 34 demonstrated that approximately 90% of the minimum dose was delivered, with fidelity improving over time from year 1 (mean 32) to year 3 (mean 35). Qualitative content analysis of 210 meetings identified 24 challenges and 15 strategies related to TCM delivery. Common challenges included engaging patients and caregivers, decreased access to post-acute services, complexity of team-based care, and limited nurse availability. Strategies to address these challenges included testing alternative approaches, building and engaging care networks, targeting patient and caregiver engagement, focusing on care coordination, and emphasizing plans of care. COVID-19 substantially disrupted trial implementation, including the withdrawal of one healthcare system, resulting in reduced enrollment (1004 of 1600 planned patient participants) and staffing delays. At trial completion, one system had integrated the Model into routine practice, while others were planning for sustainability. These findings highlight the importance of ongoing systematic assessment of both intervention delivery and trial implementation to contextualize and interpret outcomes.

**Supplementary Information:**

The online version contains supplementary material available at 10.1007/s43477-026-00234-5.

## Introduction

Caring for hospitalized older adults with complex health and social needs remains challenging within the fragmented U.S. healthcare system (Jones & Dolsten, 2024). Although evidence-based transitional care interventions improve continuity of care and health and economic outcomes, adoption has been limited, partially due to insufficient guidance on effective implementation (Gesell et al., 2021). This paper describes a multidisciplinary effort to evaluate the implementation of the Transitional Care Model (TCM), an intervention shown in multiple NIH- and foundation-funded trials to improve health outcomes of older adults transitioning from hospital to home, while reducing healthcare costs (Naylor et al., 1994, 1999, 2004, 2014, 2016; Pauly et al., 2018).

### Replicating the Transitional Care Model

The TCM is an advanced practice registered nurse (APRN)-led, team-based care management approach designed and demonstrated to reduce acute and post-acute care use and improve health outcomes of at-risk older adults transitioning from hospital to home (Naylor et al., 1994, 1999, 2004, 2014, 2016; Pauly et al., 2018). The TCM has been recognized as a “top-tiered” evidence-based solution with the potential to improve outcomes and optimize Medicare resource use if implemented on a much larger scale (Arnold Ventures, 2018). TCM relies on APRNs to provide continuity of care, trusting relationships with patients and caregivers, and coordinated acute and post-acute services (Hirschman et al., 2015). However, limited evidence guiding real-world implementation replicability continues to constrain widespread adoption (Naylor et al., 2018).

To address this knowledge gap, the Multisite Replication of the Transitional Care Model (MIRROR-TCM) randomized controlled trial (RCT) was conducted in nine hospitals situated in four healthcare systems serving diverse populations (Naylor et al., 2022). The study compared outcomes of older adults hospitalized with heart failure (HF), chronic obstructive pulmonary disease (COPD), and/or pneumonia who were discharged to home and who received the TCM protocol to a similar group who received standard discharge planning. The primary outcomes were acute care resource use and costs at 12 months post-discharge; secondary outcomes included experience with care, self-reported health, and quality of life at 90 days (clinicaltrials.gov: NCT04212962).

This paper describes a mixed-methods implementation evaluation (hereafter evaluation) conducted in parallel with the MIRROR-TCM trial to evaluate implementation of both the TCM protocol (primary aim) and trial protocols, the latter extending to sustainability of the TCM at sites (secondary aim) (Naylor et al., 2023). Prior studies established that the effective implementation and sustainability of evidence-based solutions depend on how well these interventions are adapted to local contexts (Leonard et al., 2017). A recent systematic review of contextual factors affecting diverse healthcare systems highlighted staffing, regulation, and payment policies as important influences of implementation and outcomes (Coles et al., 2020). However, few publications evaluate both the implementation of evidence-based interventions as designed and the research study’s implementation. This parallel evaluation provided a unique opportunity to explore contextual challenges encountered in conducting a complex clinical trial in diverse health systems during the first three years of the COVID-19 pandemic. The purposes of this paper are to:

1. Describe the implementation of the TCM protocol by examining the fidelity to the delivery of the intervention and contextual challenges and strategies associated with the delivery of the TCM protocol between 2020 and 2023 (primary), and.
2. Describe the implementation of the MIRROR-TCM trial by examining patient recruitment and staff hiring and training, site participation, and sustainability of the TCM at four partnering healthcare systems (secondary).

## Methods

### Design

This study utilized a convergent, parallel mixed methods design (Creswell & Plano Clark, 2018), allowing the combination of qualitative and quantitative data to form a more comprehensive understanding of a phenomenon that could not be achieved with either type of data independently. All study procedures for the evaluation were approved by the Institutional Review Board (IRB) at the University of Pennsylvania (Protocol #: 842744). Participating healthcare systems (Mathematica) had their own ethics review, approval, and consent forms for the trial.

### Framework

**Fig. 1:**
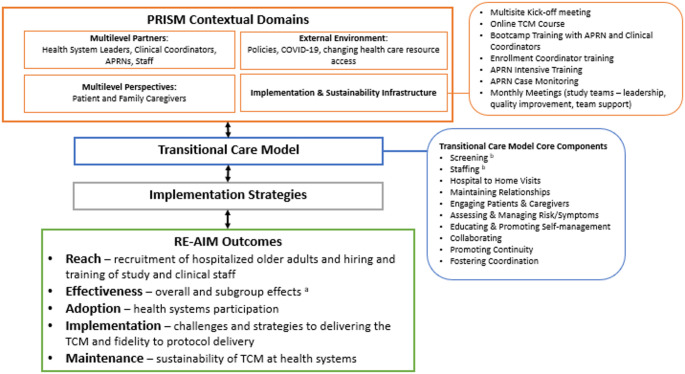
Adapted PRISM Framework for the Implementation of the Transitional Care Model. ^a^Effectiveness is not part of the outcome analysis presented here. ^b^These components were embedded in the trial design – all patients were screened using standardized procedures, and enrolled patients randomized to the TCM group were assigned an Advanced Practice Registered Nurse. Implementation strategies are available in Supplementary Information S1. (Fort et al. 2023; Naylor et al., 2023)

The Practical, Robust, Implementation, and Sustainability Model (PRISM) guided this evaluation. PRISM integrates key concepts from the Diffusion of Innovations Chronic Care Model and Institute for Health Care Improvement frameworks (Glasgow et al., 2024). Outcomes were evaluated using the Reach, Effectiveness, Adoption, Implementation, Maintenance (RE-AIM) framework as proposed by Fort and colleagues (2023). Figure 1 presents the contextual domains of PRISM in relation to the TCM and outcomes (Fort et al., 2023).

The implementation strategies (presented in Supplementary Information Table S1) were designed to launch the trial and support TCM implementation by site staff. Strategies included both virtual and in-person training and monitoring.

### Setting

The MIRROR-TCM trial was launched in hospitals situated within: Swedish Health Services (Washington); Trinity Health (Michigan), University of California San Francisco (UCSF) Health (California), and Veterans Health Administration (VHA) (Missouri and Ohio). Collectively, nine hospitals and their post-acute and community-based partners served as study sites. These hospitals served older adults in diverse geographic regions and who were underrepresented in prior TCM studies, including Veterans, rural residents, and patients who spoke little or no English.

### Sample

The study sample included older adults who met eligibility criteria and were randomized to the TCM group in the trial, and the employees of hospitals and health systems, their post-acute and community-based partners, who participated in the MIRROR-TCM trial.

#### Older Adult Sample

The original recruitment goal for the MIRROR-TCM trial was to enroll a total 1600 older adults (800 in the TCM group). Eligible patients were adults age 65 or older who lived within the health system’s service area, were hospitalized from home with HF, COPD, and/or pneumonia, communicated in a language supported by the healthcare system, and reachable by telephone. Additionally, patients had at least one other risk factor: multiple chronic conditions (5+), functional deficits or recent fall, cognitive impairment (Six Item Screener score < 3) (Callahan et al., 2002), a history of depression, a recent emergency department [ED] visit or hospitalization (within 30 days), or frequent ED visits or hospitalizations (2+) within the past six months.

Screening and enrollment data were collected by enrollment coordinators using standardized, password-protected SharePoint forms. Patients who died before discharge or within 7 days post-discharge, withdrew, or were deemed ineligible following enrollment were excluded from the final sample. Enrollment occurred between July 2020 and January 2023.

#### Healthcare System Employee Sample

Eligible healthcare system employees included leaders responsible for the TCM service line, clinical team members delivering the TCM protocol (clinical coordinators, APRNs), and research staff involved in patient recruitment and data collection.

### Data Collection

Between April 2020 and April 2023, quantitative and qualitative data were gathered to address the study aims. Guided by the PRISM framework combined with RE-AIM, data were organized into four of the five RE-AIM outcomes (see Fig. 1)–reach, adoption, implementation, and maintenance–to better explain the influence of context on the implementation of the TCM protocol within the MIRROR-TCM trial. The fifth RE-AIM outcome, effectiveness of the RCT, is not presented (Naylor et al., 2022). Below, each RE-AIM outcome is defined and operationalized. Table 1 presents the outcome, aim, data source, and data type.

**Table 1:** Outcome by aim: data sources and type of analysis

Outcome	Aim	Data source	Type of Analysis
Implementation	Primary	The fidelity score created is based on the APRN documentation of their delivery of the TCM components. A minimum intervention dose score is generated out of 38 possible TCM component elements delivered either at a specific time (e.g., visit within 24 h post-hospital discharge) or at least once during the hospitalization and/or at least once post-hospital discharge (e.g., evaluated health and/or social service needs). Refer to Table 2 for further details on the component elements.	Quantitative
		210 recordings of meetings and meeting minutes	Qualitative
Reach	Secondary	Enrollment of study participants	Quantitative
		Number of staff hired	Quantitative
		210 recordings of meetings and meeting minutes	Qualitative
Adoption	Secondary	The number and proportion of invited hospital systems that agreed to participate in the collaborative and initiate TCM	Quantitative
Maintenance	Secondary	210 recordings of meetings and meeting minutes	Qualitative

*APRN* advanced practice registered nurse, *TCM* transitional care model

### Primary Aim – Transitional Care Model Implementation

Intervention implementation was examined quantitatively and qualitatively to describe the fidelity of the TCM protocol, understand the challenges of implementing TCM, and identify strategies to address them. A minimum dose count score focused on the consistency of TCM protocol element delivery to patients assigned to the intervention arm. Guided by prior TCM research (Hirschman et al., 2017) and experts in implementation science, operational definitions of the TCM protocol core component elements were measured (Naylor et al., 2018). As part of the MIRROR-TCM trial protocol, APRNs were trained to document the delivery of TCM care components to older adults randomized into the intervention group using standardized data collection forms in a secure REDCap database (Harris et al., 2019). These forms captured each contact between the APRN and older adults, family caregivers, healthcare team members and/or community-based service providers. A minimum dose was set for each element out of a possible 38 elements that could be delivered over the course of the intervention (see Table 2).

**Table 2:** Transitional care model elements by component and minimum dose criteria for fidelity scoring

Element	Definition	Minimum dose delivered by APRN
Hospital-to-home component		
1	Visit in hospital within 24 h of enrollment	Seen 1 time during initial hospitalization within 24 h of study enrollment
2	Seen at least 1 time during the initial hospitalization	Seen 1 time during initial hospitalization
3	Daily visits during the initial hospitalization (for the first four days) and at least one visit at the hospital within 48 h of discharge	Up to 4 visits during the patient’s study enrollment hospitalization. If the initial hospitalization is less than 4 days, then seen once per day of hospitalization.
4^a^	Visit at home within 24 h of initial hospital discharge	Seen 1 time at the patient’s home
5^b^	Visited at a Skilled Nursing Facility (SNF)/Rehab within 24 h of initial hospital discharge	Seen 1 time in SNF
6^b^	Visit at home within 24 h of SNF/Rehab discharge	Seen 1 time at the patient’s home
7^c^	Visit at hospital within 24 h of readmission	Seen 1 time in hospital (after initial hospitalization)
8	Visit with Primary Care Provider/Specialist	Seen 1 time after initial hospitalization
9	A total of 9 in-person visits	Seen 9 times in person after enrollment
10	Weekly in-person visits during the first four weeks post-initial hospital discharge	Up to 4 visits post initial hospitalization — if the intervention was less than four weeks, then 1 per week of intervention
11	Weekly contacts post-discharge	The number of contacts will be equal to the number of weeks in the intervention
Promoting continuity component		
12^d^	Patient’s needs, goals, and plans of care communicated within and across care sites	Completed at least 1 time in the hospital and 1 time after initial hospitalization discharge
13^b^	Ensured SNF/Rehab staff had an updated plan	Completed at least 1 time after initial hospitalization discharge
Coordinating care component		
14^d^	Evaluated health and/or social service needs	Completed at least 1 time in the hospital and 1 time after initial hospitalization discharge
15^e^	Ensured the community agency understood health needs	15 & 16 completed at least 1 time after initial hospitalization discharge
16^e^	Assessed if agency resources were meeting patient needs	
17^d^	Priority needs and plans to address them and/or results communicated to the agency team	Completed at least 1 time in the hospital and 1 time after initial hospitalization discharge
Collaborating with patients, caregivers, and Team		
18^d^	Communicated with the hospital, health care team, and/or community agencies team to get them to work together	Completed at least 1 time in the hospital and 1 time after initial hospitalization discharge
19^d^	Collaborative design, implementation, and evaluation of care plans	19 & 20 completed at least 1 time after initial hospitalization discharge
20^d^	Care plan updated with providers	
Maintaining relationships with patients and caregivers		
21	Documentation of advocacy to achieve goals	
22	Coach the patient about preparation for visits and conversations with providers	21–23 completed at least 1 time after initial hospitalization discharge
23	Advocacy for patient’s goals and preferences with a multidisciplinary team	
Engaging patients and caregivers		
24	Patient’s goals and preferences identified	24–28 completed at least 1 time after initial hospitalization discharge
25	Patient’s goals and preferences updated or reevaluated	
26^f^	Caregiver’s goals and preferences identified	
27^f^	Caregiver’s goals and preferences updated or reevaluated	
28	Advanced planning, care preference discussion, or end-of-life care conversation	
Managing symptoms and risks		
29^d^	Symptom management needs were assessed and addressed	29 & 30 completed at least 1 time in the hospital and 1 time after initial hospitalization discharge
30^d^	Completion of clinical assessment	
31	Urgent/emergent care plan developed, updated, or reinforced	31 & 32 completed at least 1 time after initial hospitalization discharge
32	Routine symptom management plan updated and reinforced	
Educating/promoting self-management		
33	Education about the connection between the plan of care and achieving goals	
34	Education to recognize symptoms	
35	Preparation regarding symptom management skills	33–38 completed at least 1 time after initial hospitalization discharge
36	Education about how to implement an emergency plan	
37	Use of teach-back, coaching, and/or problem-solving	
38	Educated about self-care management	

TCM components of screening and staffing were included in the design of the MIRROR-TCM trial

^a^Element only applies to participants discharged directly to the home

^b^Element only applies to participants discharged directly to a skilled nursing facility (SNF) for a short stay

^c^Element only applies to participants rehospitalized

^d^Hospital assessment only applies if the patient remained in the hospital for more than 24 hours after enrollment

^e^Element only applies to participants for whom the Advanced Practice Registered Nurse made a referral to community-based services

^f^Element only applies to participants with a caregiver

Based on APRN documentation, a fidelity composite score was calculated. Each TCM element listed in Table 2 was rated as implemented (1), not implemented (0), or not applicable. Some elements required delivery within a specific timeframe (e.g., within 24 h of discharge) or at least once in the hospital and/or home setting. “Not applicable” was recorded for care in a skilled nursing facility (SNF) when patients were not discharged to a SNF. Thus, the denominator for the composite intervention fidelity score varied based on the number of possible elements that could be delivered. The possible score range was 0–38. To facilitate a comparison of fidelity scores across all older adults in the intervention group, a composite score for each patient was rescaled to reflect 38 potential items (Hanlon et al., 2023). Based on prior TCM research and application, the goal for delivering the TCM protocol with fidelity was set at achieving at least 90% of the potential 38 elements or a minimum rescaled score of 34.

Data were collected during separate, scheduled meetings of the trial research team and the site teams (e.g., leaders, clinicians, research staff) at the participating health systems. Specifically, recordings of (1) quarterly meetings with site leaders of healthcare systems and their community-based organizations, and (2) monthly meetings with site-specific clinical teams (i.e., clinical coordinators, APRNs) and research team members (i.e., site enrollment coordinators). Agendas for all meetings, co-designed with meeting participants, guided discussions regarding implementing the intervention (leadership and clinical staff meetings focused on delivering the TCM components, such as making home visits and completing clinical assessments). Meetings were also used to monitor trial progress with leaders and research staff meetings focused on admissions, screenings, and enrollments. All meetings focused on identifying implementation challenges and strategies to address them. Meetings were recorded with participants’ consent, and all recordings were uploaded to a secure, password-protected cloud folder.

### Secondary Aim – Implementation of the Trial

#### Reach

Reach, defined as the extent to which an intervention reaches the intended target population (Glasgow et al., 2024), was operationalized: first, as the number of hospitalized older adults eligible, screened, and approached for the trial, and, second, as the extent to which the MIRROR-TCM trial reached the healthcare system employees as intended, including recruiting staff, training, and participating in trial activities on the site research teams.

#### Adoption

Adoption is defined as the number of healthcare systems that are willing to initiate the trial at their organization, and the level of their success (Glasgow et al., 2024). Adoption was operationalized as the number of healthcare systems out of four that successfully completed their participation in the MIRROR-TCM trial.

#### Maintenance

Maintenance is defined as the extent to which the participating healthcare systems adopted the TCM protocol into their organizational practices (Glasgow et al., 2024). At the end of the intervention phase, maintenance was operationally assessed as a plan for sustainability after the trial. Interviews were conducted with the leaders from each participating health system that completed the trial to identify each system’s plan for adopting TCM.

### Analysis

In keeping with a parallel, convergent mixed methods design, quantitative and qualitative data were collected simultaneously and independently, and then were integrated following data collection (Creswell & Clark, 2018). The goal of these analyses was to describe the TCM protocol implementation – fidelity (e.g., how well the TCM was delivered to patients) and the challenges and strategies to implementation, and trial implementation.

#### Quantitative Analysis

Descriptive statistics were used to depict fidelity to the implementation of the TCM (indicating that a *minimum* dose of the elements was delivered as planned), reach (counts of screened and enrolled patients), and adoption (number of healthcare systems that completed the trial). For fidelity scores, means, standard deviation, medians, and ranges are presented over time based on time of enrollment: Cohort 1 (9/2020-1/2021), Cohort 2 (2/2021-1/2022), and Cohort 3 (2/2022-1/2023).

#### Qualitative Analysis

Qualitative content analysis was used to describe TCM implementation challenges and strategies to address them (Hsieh & Shannon, 2005). Codes were developed inductively to explore challenges and strategies for implementing the TCM protocol. The research team systematically reviewed recorded meetings to develop codes directly from the data. Monthly trained research assistants (RAs) reviewed recorded meetings and used initial codes to capture implementation of the TCM protocol. Codes were refined and grouped as challenges to TCM protocol implementation and strategies to address them. Data were captured on a standardized form and managed in NVivo (Jackson et al., 2019). Challenges and strategies were mapped to relevant TCM components, with year-1 data published elsewhere (McHugh et al., 2024). Initially, the study team met monthly to review the codes and definitions and subsequently met bi-monthly to review codes and definitions and examine patterns. The full codebook is available in Supplementary Information Table S2. Audit trails were used to ensure the trustworthiness and validity of the findings (Campbell et al., 2020; Vogl et al., 2019). Code counts from NVivo were used to rank themes by overall frequency and by cohort.

#### Quantitative and Qualitative Data Integration

Both qualitative and quantitative data were integrated to examine the alignment and discrepancies between intended TCM delivery, implementation challenges, and strategies used to address them. Data integration and interpretation were conducted by team members’ with methodological expertise who focused on convergence and divergence across data sources and cohort years (Campbell et al., 2020). Information gleaned from the quantitative implementation data (i.e., TCM protocol delivery fidelity) and the qualitative review of meetings (challenges and strategies to delivering the TCM protocol) was presented regularly to the sites during meetings to examine the convergence or divergence of the data. Moreover, data were mixed, using a joint display table annually to guide analysis (Younas et al., 2020) (see example in Supplementary Information Table S3). Data interpretation was routinely discussed, and the main findings were determined by consensus of the study team. Triangulation of data from multiple sources (e.g., qualitative findings from different types of meetings, quantitative screening, and enrollment data) was applied to ensure the rigor of this work (Campbell et al., 2020; Vogl et al., 2019).

## Results

Findings from qualitative and quantitative analyses of the implementation of the TCM protocol (primary aim) are presented first, followed by findings related to reach, adoption, and maintenance (secondary aim) relevant to trial implementation.

### Implementation Fidelity to the Transitional Care Model Protocol

**Fig. 2:**
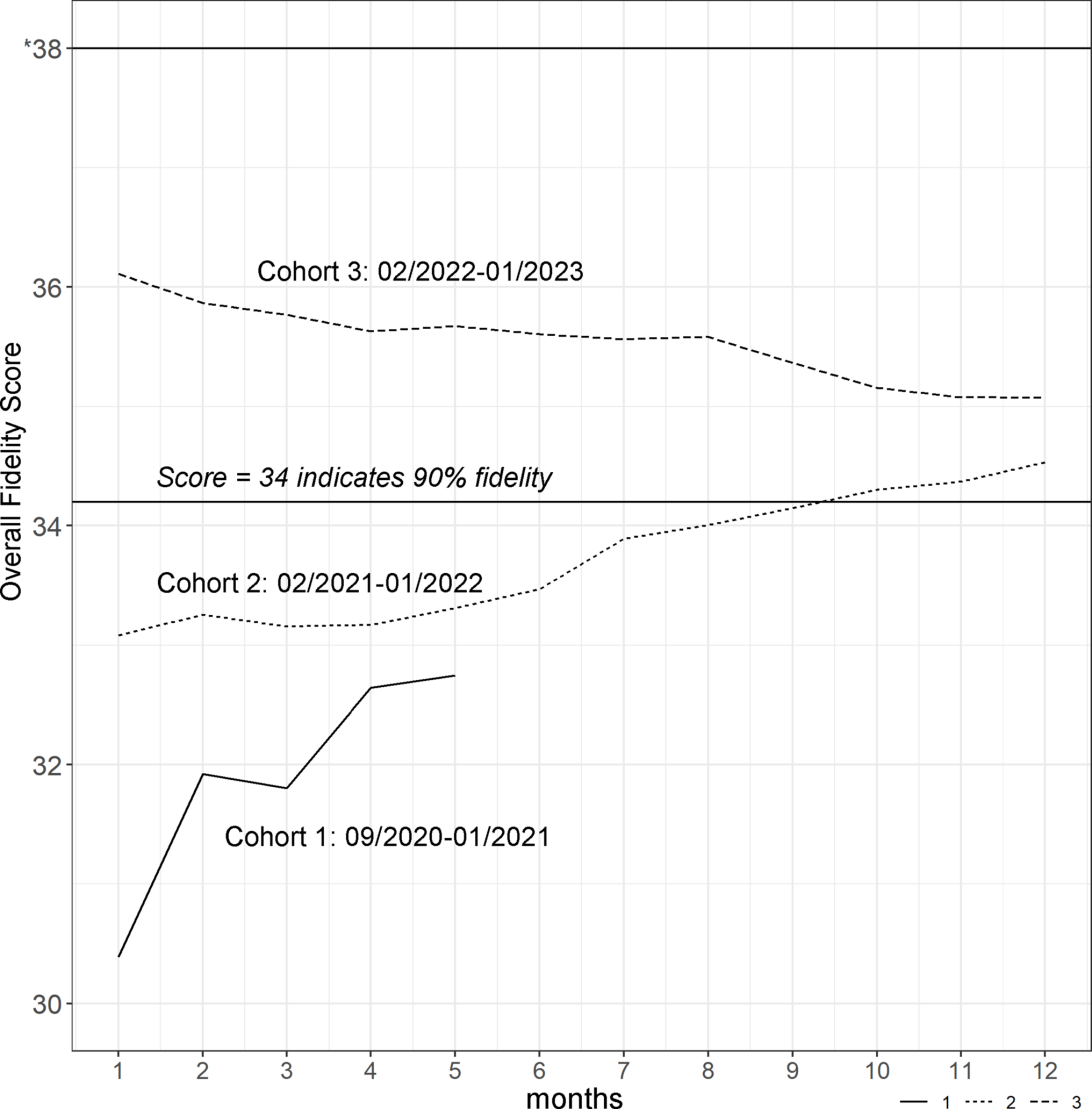
Mean transitional care model intervention fidelity score by time cohort. The X-axis represents the cohort recruitment month. The solid line (1) is Cohort 1, the fine dashed line (2) is Cohort 2, and the wide dashed line (3) is Cohort 3. The Y-axis represents the fidelity score. A score of 34 out of 38 indicates 90% fidelity to delivering the intervention as intended (100%). Data for the healthcare system that dropped out of the study is not included

Fidelity to the delivery of the TCM protocol was examined overall and over the course of the trial. Data from the APRN documentation of TCM care component delivery were used to create a minimum-dose composite fidelity score. See Table 2 for TCM component element details. The level of intervention fidelity did not meet the minimum dose for Cohort 1 but did for Cohorts 2 and 3. For Cohort 1, out of a possible score of 38 TCM elements, average intervention fidelity scores were 31.82 (standard deviation, SD ± 5.92), revealing that Cohort 1, on average, received 87.3% of the TCM component elements, lower than the planned rate of 90% (see Fig. 2).

However, for Cohort 2, the average fidelity score was 33.98 (SD ± 3.68), and for Cohort 3 it was 34.79 (SD ± 2.76), indicating that both cohorts received approximately 90% of the TCM protocol elements. Intervention fidelity scores by healthcare system and cohort are shown in Supplementary Information Figure S1.

### Implementation of the Transitional Care Model Protocol: Challenges and Strategies

Concurrent with the quantitative analysis of implementation fidelity from 2020 to 2023, described above, qualitative data were used to describe the challenges associated with delivering the TCM protocol and the strategies to overcome them. Qualitative analyses of data collected over 35 months included 210 recorded meetings (52 site leadership meetings, three community partner meetings, 140 clinical team meetings, and 15 research team meetings). Analyses revealed 24 unique codes related to challenges in implementing the TCM protocol, and 15 unique codes related to strategies to overcome these challenges (see Table 2 for the most common codes and definitions and see Supplementary Information Table S2 for the full codebook of challenges and strategies).

#### Implementation Challenges

**Fig. 3:**
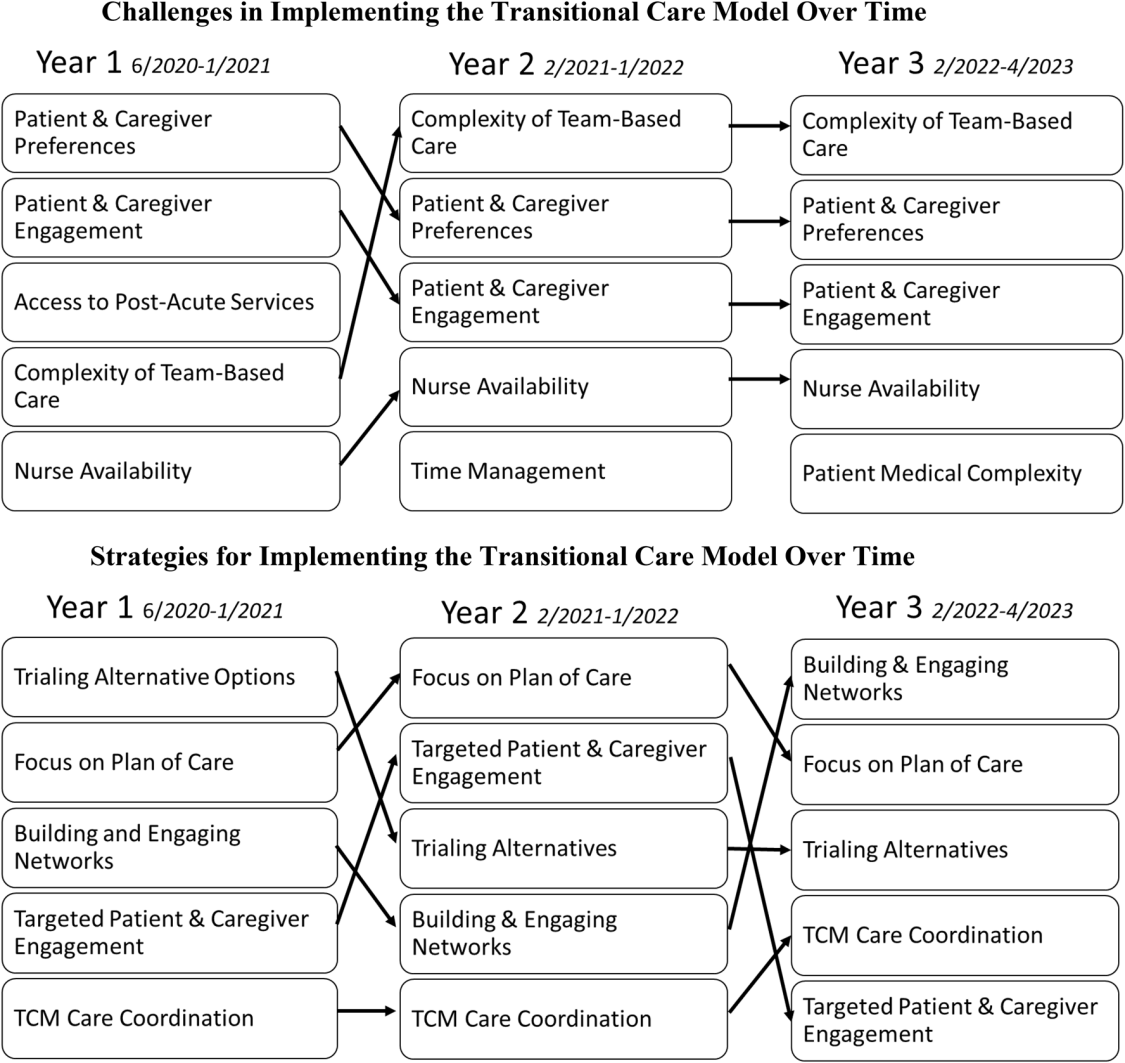
Challenges and strategies for implementing the transitional care model over time. year 1 recordings started in June 2020. this includes the one healthcare system that dropped out of the trial since recruitment began in July 2020

The most commonly coded challenges to implementing the TCM protocol were (1) patient and caregiver preferences, (2) patient and caregiver engagement, (3) access to post-acute services, (4) complexity of team-based care, and (5) nurse availability; see Fig. 3 and Table 3.

Clinical staff delivering the intervention reported that providing care consistent with *Patient and Caregiver Preferences* made it challenging to implement the defined visit expectations with the patient and their family caregiver(s). For example, patients selecting specific days for visits or to avoid visits on holidays hindered maintaining fidelity (e.g., unable to schedule or complete timely visits). Similarly, *Patients and/or Caregivers who were not Engaging* with the Intervention (e.g., “not answering calls”) created additional challenges to maintaining fidelity. An APRN stated, “I have been unable to complete timely visits.” These challenges were attributed to COVID-19 and other factors. For example, older adults and their caregivers in Cohort 1 declined needed post-acute care services, including visits to primary care providers [PCP] or specialists, or home visits by physical therapists, due to a fear of exposing themselves or family members to COVID-19. The *Complexity of Team-Based Care* also compromised intervention fidelity due to challenges with team member communication. APRNs described instances when hospital teams were unable to anticipate the discharge date, thereby reducing clarity about the start of post-discharge care. An APRN stated, “Discharge timing is not clear.” In other cases, a barrier to teamwork was scheduling follow-up appointments. An APRN stated, “PCP appointments are not available.” Finally, when delays in hiring APRNs occurred, *Nurse Availability* was a challenge attributed to COVID-19-related hiring freezes or to APRNs being deployed to other positions. This barrier required that study APRNs visit some older adults outside of the expected 24-hour window defined by the TCM protocol. Throughout the study, COVID-19 infections or exposure among older adults, their family members, and APRNs contributed to delays in delivering the TCM protocol both in the hospital and in the home.

In the second and third years, APRN *Time Management* emerged as a key factor in completing tasks, specifically related to travel time, transportation, and traffic, as well as finding time to document patient care plans. All sites reported an increase in this challenge as caseloads increased. By Cohort 3, the *Complexity of Team-Based Care* remained the number one challenge to intervention fidelity. Even as COVID-19 admissions slowed and healthcare systems began to return to a new normal, these challenges continued. *Patient Medical Complexity*, which consistently ranked just below the top five challenges for Cohorts 1 and 2, emerged as one of the top five challenges for Cohort 3. The higher medical complexity of older adults in this study, compared to earlier TCM studies, complicated their care, especially managing multimorbidity and co-occurring symptoms of insomnia, fatigue, depression, and geriatric syndromes, such as frailty and fall risk (see Table 3).

**Table 3:** Challenges and strategies codes related to implementing the transitional care model

Challenges Codes	Definitions
Patient and caregiver preferences	Patient and/or caregiver preferences (e.g., preferring specific timing of visits, female providers, or avoiding visits on holidays) may direct care in a way that works for them but also pose challenges to maintaining fidelity (e.g., being unable to schedule or complete timely visits). Patients and caregivers are engaged with the intervention but express preferences regarding how it is delivered, specifically tailored to their individual situations and needs.
Patient & Caregiver Engagement	Patients and/or caregivers are not engaging in the intervention (e.g., not answering calls, lack of engagement), which challenges delivering the intervention as intended (e.g., being unable to schedule or complete timely visits; declining needed services).
Access to post-acute services	Patients and/or families decline needed non-TCM post-acute care services (e.g., in-person primary care or specialist follow-up visits, physical therapy).
Complexity of team-based care	Challenges with how care is provided by others besides the APRN negatively impact fidelity, typically because of timing and/or delays and challenges with communication and coordination (e.g., the patient is receiving tests in the hospital and unavailable for an APRN visit, primary care provider appointments are not available, discharge timing is unclear, variable, or not communicated).
Nurse Availability	APRN availability impacts the ability to complete certain aspects of care (e.g., attending a primary care provider visit, conducting in-person visits) for various reasons (e.g., illness, vacation, other duties).
Time management	Challenges include completing all tasks in a timely manner (e.g., finding time to document, managing a schedule, managing a full caseload, managing geographically distant patients, and time-intensive travel).
Patient medical complexity	Patients significant medical complexity makes managing their care very challenging overall (e.g., multimorbidity; symptom clusters like insomnia, fatigue, and depression; unmanaged symptoms; geriatric syndromes like frailty and fall risk; unable to wear a mask because it worsens shortness of breath; COVID-19 complicated by multi-morbidities, poor living situation, strained relationships with caregivers, issues making the APRN feel unsafe in the patients home).

Strategies codes	Definitions
Trialing alternative options	Exploring alternative options to current challenges by identifying supporting systems and workarounds (e.g., telehealth or phone visits or visits through the window in place of in-person visits; reach out to other established care providers to contact the patient if they are not responding to calls, change visit schedule to meet patient/caregiver preferences and document reason, schedule a phone visit if unable to complete in-person visits).
Focus on plan of care	Strategies for establishing an effective plan of care, including assessing community care needs and linking community services to goal-concordant care plans.
Building & Engaging Networks	Build and engage networks and relationships across healthcare and community settings to address barriers, such as developing relationships with local rehabilitation facilities and providers, contacting payers for resources like iPads, and coordinating with leadership at post-acute care facilities for site access.
Targeted patient and caregiver engagement	Recommendations for effective communication with patients and caregivers (e.g., scheduling in-person visits, guidelines on the frequency of phone calls, sending letters if unable to reach a patient by phone, and clearly communicating the goals of the visit).
TCM care coordination	Strategies for communicating and coordinating care within and across healthcare systems (e.g., setting up visits, joining visits in person, making sure a plan of care is carried out, finding out necessary information from specialists like prognosis) through partnerships between healthcare professionals, the TCM APRN, and patients in designing plans of care and ensuring effective implementation of these plans.
*TCM* transitional care model, *APRN* advanced practice registered nurse	

#### Strategies to Overcome Challenges to Implementation

Healthcare system leaders, clinical coordinators, and TCM APRNs identified several strategies to address challenges to achieving high intervention protocol fidelity. The most commonly coded strategies for implementing the TCM protocol were (1) trialing alternative options, (2) building and engaging networks, (3) targeting patient and caregiver engagement, (4) enhancing TCM care coordination, and (5) focusing on plans of care (see Fig. 3). Most commonly, APRNs described *Trialing Alternative Options*, such as using telehealth technology or conducting window visits in place of in-person visits due to COVID-19 restrictions. APRNs reported the need for *Building and Engaging Networks* across health care and community settings to address access challenges. They also reported a larger-than-expected need to find telehealth resources. For example, APRNs working in the VA set up a “Digital Divide Consult,” a program that assesses the need for internet access or an internet-connected device. Other strategies to engage other networks included contacting payors for iPads and coordinating with leaders at post-acute care facilities to safely gain access to sites.

Healthcare system staff and APRNs made several recommendations for effective communication with older adults and their family caregivers. They described *Targeted Patient and Caregiver Engagement Strategies*, that placed greater emphasis on strengthening patient engagement such as trying to schedule visits while visiting the patient, calling at different times of day, sending letters (if unable to reach a patient or their family member by phone), using translator services in and out of the hospital, tailoring communication to the individual, and rooting communication in the older adult’s goals and values. Moreover, APRNs used strategies for *coordinating care* within and across healthcare systems. They also coordinated and attended appointments, ensured care plans were implemented, gathered specialist information, and supported care planning with older adults and family caregivers. Finally, strategies for establishing an appropriate *plan of care* included allocating extra time to assess the older adults’ community support needs, linking community services to goal-concordant care plans, and coordinating follow-up care.

### Secondary Aim

The data presented below explores the outcomes of reach, adoption, and maintenance.

#### Reach

*Older Adult Sample. *Reach was assessed by the number of eligible patients identified, screened, and enrolled. The MIRROR-TCM trial originally aimed to enroll 1600 older adults (400 per healthcare system) over 18 months, with enrollment beginning May 2020 and concluding in October 2021. However, COVID-19 delayed healthcare system launches, reduced the eligible patient pools, and extended recruitment through January 2023. Although the team anticipated that 30% of hospital admissions would be eligible, only 7.1% (3,053/42,794) were potentially eligible, and 1665 (54.5%) met eligibility after screening. Between July 2020 and January 2023, 1,004 participants were enrolled, with enrollment rates varying by site (Site A: Eligible, *n* = 663; Enrolled, *n* = 262, 39.5%; Site B: Eligible, *n* = 370; Enrolled, *n* = 245, 66.2%; Site C: Eligible, *n* = 563; Enrolled, *n* = 454, 80.6%; Site D: Eligible, *n* = 69; Enrolled, *n* = 42, 60.9%). Common reasons for nonparticipation included lack of interest in research or the study (20.3%), perceived lack of need (14.9%), time constraints (9.4%), and early hospital discharge (6.7%). Of the 1004 enrolled patients, 26 withdrew, 19 died before hospital discharge or immediately after discharge (< 7 days), nine were determined ineligible, and 42 were at a site that withdrew (see Adoption). Thus, the final sample included 908 patients (443 TCM group; 465 Control group), representing 57% of the planned sample. Despite lower enrollment, the TCM and Control groups were well-balanced on baseline characteristics (see Supplementary Information Table S4 for patient demographics).

*Healthcare System Employee Sample.* Although staff reach was ultimately achieved, COVID-19 significantly delayed implementation. The study originally planned a three-month launch period (March-May 2020, see Supplementary Information Table S1), but actual roll-out ranged from five to 11 months across healthcare systems (1.5 to 3.5 times longer than expected) due to pandemic-related disruptions, including delayed hiring, staff turnover, and reliance on part-time APRN models at some sites. For example, the study design specified an in-person training event with all APRNs (*n* = 6) and clinical coordinators (*n* = 4). However, this training was canceled due to the issuance of COVID-19-related community mitigation policies implemented in March 2020. In addition, while each site was committed to hiring two APRNs, Site A took longer to find and hire a second APRN, Site B had APRN turnover during the trial, and Site C had two hospitals in two different states and only hired one APRN full-time and tried different strategies for part-time APRN staffing to support the full-time APRN. The study team adapted by pivoting to online training to accommodate pandemic-related constraints. Pandemic-related hiring delays postponed training and launch dates, slowing progress toward study benchmarks. In response, the team successfully shifted to virtual group and site-specific training, but these delays also necessitated changes in implementation data collection, including more frequent meetings with healthcare system leaders and staff.

### Adoption

Three of four healthcare systems adopted the MIRROR-TCM trial protocol as intended. One healthcare system (Site D) stopped enrollment in January 2021, after 42 enrollments, citing COVID-19-related barriers, including fewer eligible patients and staffing challenges. The site completed the study for their enrolled patients. The remaining three healthcare systems completed the trial as planned.

#### Maintenance

Leadership and staff in three healthcare systems that adopted the intervention committed to sustaining TCM delivery after study completion, expressing optimism about its value for older adults. At Site A, team members emphasized better coordination for patients discharged home, particularly addressing social determinants of health, home health care support, and communication with PCPs. Site A implemented a Post-Acute Transition Care program for its Accountable Care Organization population and adapted the TCM to utilize a registered nurse (RN) who engages patients in the hospital before transfer, coordinates care across settings, and conducts home visits. At Site B, population health department staff reported commitment to a “TCM 2.0” program, though implementation was delayed due to financial constraints. Site C proposed new or revised TCM programs across two hospitals: one planned integration into a Geriatric Transitional Care Program with a comprehensive geriatric assessment during the inpatient hospitalization, transitional care, and follow-up with the PCPs, while the other launched a modified TCM 2.0 program that removed age restrictions, reduced diagnosis limits, and expanded telehealth use to widen their geographical reach.

## Discussion

This study examined the challenges and strategies involved in implementing a multicomponent transitional care intervention in the MIRROR-TCM trial and identified factors influencing reach, adoption, and maintenance. The findings offer insight into implementing interventions in dynamic, diverse healthcare settings and have several implications.

Overall, COVID-19 was a major contextual factor affecting the delivery of the TCM protocol in the MIRROR-TCM trial. Although most implementation strategies required modification (Table 1), the full impact on implementation fidelity remains unknown. Similar adaptations were reported in a transitional care program that shifted from in-person to virtual delivery due to the pandemic (Takahashi et al., 2024). Together, these findings underscore the importance of ongoing monitoring of multicomponent interventions to identify system-specific challenges, find solutions, and ultimately improve fidelity over time.

First, a convergent, parallel mixed-methods design incorporating both quantitative and qualitative data revealed a holistic picture of the challenges and strategies needed to implement the multicompetent TCM protocol and the MIRROR-TCM trial in the context of COVID-19. Concurrent quantitative and qualitative analyses captured pandemic-related impacts on intervention delivery (implementation), sample recruitment and staffing (reach), healthcare system participation (adoption), and sustainability planning (maintenance). Integration of healthcare system leadership, APRNs, and staff perspectives, along with fidelity scores, demonstrated convergence between perceived protocol implementation challenges and strategies. The mixed-methods approach also clarified why three of the four healthcare systems fell short on enrollment targets (400 per healthcare system). Estimated enrollment projections were derived from pre-pandemic hospitalization patterns for targeted diagnoses, which were significantly disrupted between July 2020 and January 2023 by the pandemic (Bozkurt et al., 2025; Shoaib et al., 2021). People avoided seeking any kind of care during the pandemic (Anderson et al., 2021), resulting in fewer hospital admissions for non-COVID-19 reasons. The prioritizing of admissions of COVID-19 patients (Birkmeyer et al., 2020), coupled with involuntary delays due to availability constraints and patient avoidance of care seen across the U.S. (Bronchetti et al., 2023; Byrnes et al., 2021), influenced the sample size of this study and potential implications for the overall trial outcomes, which have not yet been reported.

Second, using PRISM as a framework is a feasible approach for understanding how contextual factors influence implementation outcomes, which is essential for interpreting the anticipated MIRROR-TCM trial findings. For example, although the original staff training plan was disrupted, the study team adapted and successfully launched the trial with a modest delay. In contrast, healthcare systems faced greater constraints early in the pandemic (March-May 2020), making it more difficult for them to pivot. Healthcare systems’ responsiveness to urgent community needs, along with hiring freezes and policy changes, limited their capacity to initiate the trial on the original timeline. Each healthcare system was committed to participation, and the study team’s flexibility in adapting to each health system’s unique circumstances was essential.

Third, regular meetings with the healthcare system leadership enabled assessment of adoption and provided insight into why one site withdrew from the trial. As noted in the results, at the start of year two (February 2021), hospital leaders and site staff reported implementation challenges they were unable to overcome, including COVID-19-related restrictions on patient access, increasing patient complexity, and concerns about low recruitment. Review of qualitative meeting data with this site’s leadership, clinical, and research teams revealed that this site faced challenges similar to those encountered by other sites during the same period; however, multiple changes in leadership, including the loss of the key champion of the program, likely contributed to the site’s inability to continue.

Finally, this study underscores the importance of monitoring implementation fidelity throughout a trial. Consistent with a Type 1 Hybrid design, the MIRROR-TCM trial prioritized testing TCM effectiveness while concurrently examining implementation processes (Curran et al., 2012). Fidelity varied across cohorts, largely reflecting the fact that a significant historical event (COVID-19 pandemic) impacted how the APRNs delivered the intervention in this trial. Although overall improvements in TCM delivery were observed over time (Fig. 2), early fidelity challenges differed across study settings (see Supplementary Information for additional figure) and were influenced by pandemic-related disruptions, start-up challenges, APRN documentation errors, or combinations of these factors. Improvements in fidelity likely reflected both increased familiarity with the intervention and ongoing quality-improvement efforts that strengthened APRN documentation practices. For instance, a dip in fidelity observed in July 2021 coincided with a national surge in COVID-19 hospital admissions (IHME, 2021). These findings highlight the importance of accounting for contextual influences and engaging local staff members in iterative improvement processes, consistent with prior implementation research (Fakha et al., 2022; Leeman et al., 2024; Zhu et al., 2023). Thus, a key implication is that regular assessment of contextual factors and responsive strategies is vital.

### Limitations

This study has notable limitations. First, although the study was designed to be implemented across four healthcare systems, only three fully adopted the intervention, limiting generalizability. However, the diversity of the participating healthcare systems, including geographic dispersion and diverse patient populations, strengthens the relevance of the findings.

Second, the qualitative data used to identify implementation challenges and strategies were collected for different purposes and across different meeting types, introducing potential variability in coding. Leadership, community partners, clinical teams, and study staff meetings differed in participants and objectives. For example, leadership meetings were commonly used to discuss contextual challenges specific to the delivery of the TCM. In contrast, clinical calls were designed to support the site APRNs and clinical coordinators in delivering the TCM per the intervention protocol and to discuss clinically challenging cases. However, directed content analysis revealed consistent themes across meeting types, suggesting robustness of findings.

Third, fidelity was measured as delivery of a minimum dose of TCM components, which does not capture variation in patient need or engagement. Most participants received more than the minimum dose of the TCM, though not every participant needed the same amount of each component. While fidelity scores improved over time, increases may partially reflect improved APRN documentation rather than changes in patient behavior or engagement.

Assessing the delivery of multicomponent evidence-based interventions, such as the TCM, is necessary but challenging (Ginsburg et al., 2021). Fidelity challenges were exacerbated by COVID-19, including using the training protocol to prepare APRNs, achieving intervention fidelity, and the limited availability of PCPs, home care workers, and SNF staff after hospital discharge. In addition, TCM fidelity is operationalized using a large group of components and their elements (Table 2), which supports detailed implementation management but complicates assessment of overall fidelity. Consistent with Ginsberg (2021), these findings highlight the difficulty of measuring fidelity of multicomponent interventions.

Finally, COVID-19 and healthcare system-level implementation challenges necessitated real-time adaptations to the TCM protocol, complicating reliable measurement of fidelity. Consistent with Ginsberg (2021), these findings highlight the need for implementation research to improve fidelity assessment strategies during intervention delivery. Nevertheless, this study provides insights into how intervention fidelity evolves and offers an example for monitoring complex interventions over time. While many implementation challenges and strategies were consistent, some strategies addressed multiple challenges, and their effectiveness could only be inferred from observed changes in fidelity scores. The pandemic also disrupted implementation in anticipated and unanticipated ways, limiting generalizability. A strength was the ability to evaluate a large-scale implementation under disruption, offering insights for future research.

## Conclusion

The study highlights key lessons in monitoring study and intervention implementation. Despite significant challenges - many exacerbated by the COVID-19 pandemic - the MIRROR-TCM randomized controlled trial identified effective strategies to address implementation barriers. Integrating qualitative and quantitative data offered a comprehensive picture of implementation complexity and provides valuable guidance for future efforts to improve transitional care for older adults.

## Supplementary Information

Below is the link to the electronic supplementary material.

Supplementary Material

## Data Availability

**The data supporting the findings of this study are available from the authors upon request and subject to a data sharing agreement with the University of Pennsylvania.**.
