# Supplementary material for: Implementation Fidelity of the Transitional Care Model: A Mixed Methods Study
Source: Glob Implement Res Appl. 2026 May 9;6(3):455–70. doi: 10.1007/s43477-026-00234-5 (PMC13403246; doi:10.1007/s43477-026-00234-5)
Supplement: Supplementary file 1 [file 43477_2026_234_MOESM1_ESM.docx]

**Supplementary Information**

**TITLE**: Implementation Fidelity of the Transitional Care Model: A Mixed Methods Study

**JOURNAL**: Global Implementation Research and Application

**SUBMISSION ID:** 3832dc71-da77-4fec-91d2-fa8880e45192 v.2.0

**Table S1**

*MIRROR-TCM PRISM Implementation Strategies, Definitions, and Program Month at Launch of Study*

| **PRISM Implementation Strategies** | **Definition** | **Planned Month** |
| --- | --- | --- |
| Multisite Kick-off meeting | All sites engaged in virtual meetings for 90 minutes | 1 |
| Online TCM Course | Completion of the online Transitional Care Model (TCM) training course (25 educational hours/asynchronous) | 1 |
| TCM Bootcamp Training | In-person meeting for intensive 2-day training bringing all the site APRNs and Clinical Coordinators together with the Penn Coordinating Center team | 2 |
| Enrollment Coordinator training | Virtual training over four hours on the research design, screening and enrollment protocols, and study documentation forms. | 2 |
| APRN 1:1 Training | Review of APRN competencies and development of tailored experiences. Dedicated time with TCM clinical experts. | 2 |
| APRN Case Monitoring | A TCM clinical expert reviews the documentation of the first five assigned patients. | 3-18 |
| Monthly Meetings (Enrollment Coordinator; All Site APRNs; Site Leadership; Site-specific Quality Improvement/Support) | Each month, the site teams meet with TCM clinical and research experts to monitor adherence to the protocol and the implementation of TCM. | 2-18 |

*Note*: APRN = Advanced Practice Registered Nurse.

**Table S2**

*Definitions of All Challenges and Strategy Codes Related to Implementing the Transitional Care Model Intervention Protocol*

| **Challenges Codes, N=24** | **Definitions** |
| --- | --- |
| Patient and Caregiver Preferences | Patient and/or caregiver preferences (e.g., preferring specific timing of visits, female providers, or avoiding visits on holidays) may direct care in a way that works for them but also pose challenges to maintaining fidelity (e.g., being unable to schedule or complete timely visits). Patients and caregivers are engaged with the intervention but express preferences regarding how it is delivered, specifically tailored to their individual situations and needs. |
| Patient & Caregiver Engagement | Patients and/or caregivers are not engaging in the intervention (e.g., not answering calls, lack of engagement), which challenges delivering the intervention as intended (e.g., being unable to schedule or complete timely visits; declining needed services). |
| Access to Post-Acute Services | Patients and/or families decline needed non-TCM post-acute care services (e.g., in-person primary care or specialist follow-up visits, physical therapy). |
| Complexity of Team-Based Care | Challenges with how care is provided by others besides the APRN negatively impact fidelity, typically because of timing and/or delays and challenges with communication and coordination (e.g., the patient is receiving tests in the hospital and unavailable for an APRN visit, primary care provider appointments are not available, discharge timing is unclear, variable, or not communicated). |
| Nurse Availability | APRN availability impacts the ability to complete certain aspects of care (e.g., attending a primary care provider visit, conducting in-person visits) for various reasons (e.g., illness, vacation, other duties). |
| Time Management | Challenges include completing all tasks in a timely manner (e.g., finding time to document, managing a schedule, managing a full caseload, managing geographically distant patients, and time-intensive travel). |
| Patient Medical Complexity | Patients significant medical complexity makes managing their care very challenging overall (e.g., multimorbidity; symptom clusters like insomnia, fatigue, and depression; unmanaged symptoms; geriatric syndromes like frailty and fall risk; unable to wear a mask because it worsens shortness of breath; COVID-19 complicated by multi-morbidities, poor living situation, strained relationships with caregivers, issues making the APRN feel unsafe in the patients home). |
| Social Determinants of Health | Factors related to the social determinants of health (e.g., finances, employment, social support, social isolation, safety) that cause challenges with implementation of the TCM (e.g., patient cannot afford medications, cluttered and dirty environment putting the patient at risk for falls). |
| Intervention Learning Curve | APRNs' knowledge about an aspect of the TCM (e.g., expected timing of visits, how long intervention should continue, strategies for scheduling visits) impacts delivering the intervention as intended (e.g., the length of time between visits is too long, patients receive intervention for too long). |
| COVID-19 Related Limited Post-Acute Resources | APRN identifies the needed service, but it is not available or delayed in scheduling services (e.g., home care has a 30 day wait; supply chain issues; no available rooms in skilled nursing facilities). |
| Post-COVID Delays in Care | Post acute care services that do exist are taking longer to begin or having longer delays between visits |
| Need for Goal Alignment | Goals are not aligned with the patients’ needs (e.g., patient with advanced condition does not have an Advanced Directive, patient has had a health change following a hospitalization and needs to update goals). |
| COVID Exposure | A possible, probable, or confirmed COVID infection or exposure for either the APRN, patient, and/or family that causes a challenge to delivery of in-person services in the site of healthcare delivery (e.g., hospital, post-acute facility, ambulatory care, or home). |
| Medication or Equipment Issues | There is a problem with medication or equipment (e.g., medication and/or equipment are incorrect, broken, difficult to administer, causing side effects) that puts the patient at risk |
| Identifying and Evaluating Best Fit | Identifying the best fit for the patient needs with the available community resources (e.g., determining next site of care that fits patient's care needs and insurance requirements and has availability, finding a service that meet patient preference - meal delivery or home delivery that patient likes, providers that are within driving distance). |
| Nurse Distress | APRNs express feelings of distress that could lead to burn out (e.g., inability to "close the door" at the end of the workday, feeling deflated after caring for a challenging patient). |
| System Onboarding | Onboarding requires a significant amount of time and effort (e.g., frequent meetings, EMR training, telehealth training). |
| Challenges with Equipment for Safe Care | Challenges with equipment that would make having a visit feasible or safe (e.g., PPE, telehealth equipment). Initially, this was just having access to equipment, but later there were issues using the equipment (e.g., "there is too much noise in the hospital room to hear the patient on the tablet"). |
| Caregiver Limitations | Caregiver involvement is limited because of COVID (e.g., unable to see the patient in-person because of restrictions or risk, caregivers are overwhelmed with COVID-related stressors). |
| Virtual Visits | Challenges related to visits being virtual, rather than in-person (e.g., virtual rounding, virtual shadowing, many collaborating health care provider visits are virtual). |
| COVID Policy Changes | Policy changes because of COVID (e.g., non-essential research on hold, research personnel not allowed in patient rooms) that lead to changes in the way care is delivered or research is conducted (e.g., inability to consent in-person) |
| Challenges Establishing Trust | Challenges establishing trust with patients and/or families (e.g., PPE and virtual visits create physical barriers to connection, APRNs could expose patients and families to COVID). |
| Uncertainty | There is uncertainty in information that could inform goals (e.g., unclear benefits of a procedure, unclear designation of proxy). |
| Changing Guidelines | There is uncertainty about new guidelines for managing symptoms and risks related to COVID. |

| **Strategies Codes, N=15** | **Definitions** |
| --- | --- |
| Trialing Alternative Options | Exploring alternative options to current challenges by identifying supporting systems and workarounds (e.g., telehealth or phone visits or visits through the window in place of in-person visits; reach out to other established care providers to contact the patient if they are not responding to calls, change visit schedule to meet patient/caregiver preferences and document reason, schedule a phone visit if unable to complete in-person visits). |
| Focus on Plan of Care | Strategies for establishing an effective plan of care, including assessing community care needs and linking community services to goal-concordant care plans. |
| Building & Engaging Networks | Build and engage networks and relationships across healthcare and community settings to address barriers, such as developing relationships with local rehabilitation facilities and providers, contacting payers for resources like iPads, and coordinating with leadership at post-acute care facilities for site access. |
| Targeted Patient and Caregiver Engagement | Recommendations for effective communication with patients and caregivers (e.g., scheduling in-person visits, guidelines on the frequency of phone calls, sending letters if unable to reach a patient by phone, and clearly communicating the goals of the visit). |
| TCM Care Coordination | Strategies for communicating and coordinating care within and across healthcare systems (e.g., setting up visits, joining visits in person, making sure a plan of care is carried out, finding out necessary information from specialists like prognosis) through partnerships between healthcare professionals, the TCM APRN, and patients in designing plans of care and ensuring effective implementation of these plans. |
| APRN Time Management Strategies | Strategies for managing and prioritizing time (e.g., document immediately after visit, round at hospitals in the morning, cluster patients in geographic areas, plan flex time for emergencies, have a system for organization, pack a bag for quick access to resources, use time with lower caseloads effectively, advocate for protected time). |
| Normalization & Validation | Penn Team or APRNs offer validation and normalization that some outcomes are inevitable and appropriate (e.g., daily hospital visits are not always required, patients and families don't always respond to phone calls). |
| Anticipation of Patient and Caregiver Needs | Anticipate and address needs that may lead to negative patient outcomes later (e.g., set up telehealth for those who need it; make a plan for if the patient is discharged rapidly or over the weekend; increase the frequency of visits if the patient's health condition deteriorates; prepare for discharge from TCM - transition to health promotion, trial reduction of visits, create a safety net). |
| Optimizing Communication with Members of the Team | Recommendations for effective communication across settings (e.g., call for daily updates), with patients and caregivers (e.g., organizing notes so the most important information is at the top, determining which communication system providers use, recommendations for frequency and type of communication). |
| Clarifying or Adapting TCM Processes | Clarify protocols or adapting TCM processes (e.g., can mark plan as re-evaluated if urgent/emergent plan wasn't necessary). |
| Anticipation of Staffing Needs | Anticipate and address needs that may lead to negative patient outcomes later (e.g., plan for weekend discharges ahead of time, discharge patients in a timely fashion to decrease caseload, determine feasible plan for coverage). |
| Addressing and Setting Goals | Strategies for addressing goals (e.g., focus on positives, take "baby steps", break big goals into smaller goals, be present, involve caregivers, "pick your battles", "do the best you can") and setting goals (e.g., goals should be patient-directed, involve caregivers). |
| Establishing Trust | Develop the patient-provider relationship through strategies to build trust (e.g., focus on the relationship). |
| Personal Safety | Strategies for keeping the APRN safe in a situation where he or she is at risk for COVID exposure (e.g., PPE, testing, screening, and gathering information). |
| Penn Team Collaboration | Coordinate and/or collaborate with a member of the study/research team (e.g., Penn Team to review enrollment and answer questions, staff across sites collaborate to learn from one another). |

*Note*: TCM = Transitional Care Model; APRN = advanced practice registered nurse

**Table S3**

*Example of Study Year 2 Structured Mixed Methods Joint Display Table*

| **Hospital-to-Home**  **Fidelity Results** | | **Qualitative Content Analysis**  **Non-COVID** | **Qualitative Content Analysis**  **COVID** | **Convergent Interpretation**  *examples* |
| --- | --- | --- | --- | --- |
| **Element** | **%** | **Challenges**   - Intervention Learning Curve - Nurse Availability - Patient and Caregiver Preferences - Complexity of Team-Based Care - Patient and Caregiver Engagement   **Strategies**   - Normalization & Validation - Targeted Patient and Caregiver Engagement - Trialing Alternative Options - Anticipation of Patient and Caregiver Needs - TCM Care Coordination - Building and Engaging Networks - Clarifying or Adapting TCM Processes - Penn Team Collaboration | **Challenges**   - COVID Exposure - COVID Policy Changes - Access to Post-Acute Services - Challenges with Equipment for Safe Care   **Strategies**   - Building and Engaging Networks - Personal Safety - Anticipation of Patient and Caregiver Needs - Trialing Alternative Options | - While most Hospital-to-Home visit patterns improved from Y1 to Y2, new non-COVID-related challenges were identified in Y2 relating to general implementation of the Hospital-to-Home component.   - *Complexity of Team-Based Care* *-* “…there are weeks-long waits to see PCP’s and specialists after hospital discharge” [Clinical Team Call] - Despite overall improvements since Y1, challenges around Patient and Caregiver Engagement, Nurse Availability, and Challenges with Care Provision appear to be driving lower fidelity to timed visits across all sites   - Access to Post-Acute Services *Patient and Caregiver Engagement*- “Patient canceled first home visits with APRN because he needed to take his spouse to an appointment.” [Clinical Team Call]   - *Nurse Availability*- “Discharges fall on the weekend when there is only one APRN available; prevents a visit at home within 24 hours of hospital discharge.” [Clinical Team Call]   - *Complexity of Team-Based Care-* “APRNs are not getting notified that their patients are being discharged from SNFs.” [Clinical Team Call] - All sites have developed new non-COVID related strategies to address challenges impacting Hospital-to-Home fidelity in Y2. It is possible that the long-term impact of some of these new strategies is not yet seen in the fidelity data.   - *Building and Engaging Networks*- “APRN tries to reach out to outside providers for updates; some providers are very cooperative in notifying discharge but others don’t respond.” [Clinical Team Call] |
| 1 Visit in hospital within 24 hours of enrollment | 97% |  |  |  |
| 2 Seen at least 1 time during index hospitalization | 99% |  |  |  |
| 3 Daily visits in hospital | 90% |  |  |  |
| 4 Visit at home within 24 hours of hospital discharge (index)* | 52% |  |  |  |
| 5 Visit at SNF/Rehab within 24 hours of hospital discharge (index) | 57% |  |  |  |
| 6 Visit at home within 24 hours of SNF/rehab discharge | 35% |  |  |  |
| 7 Visit in hospital within 24 hours of readmission | 54% |  |  |  |
| 8 Visit with Primary Care Provider/ Specialist | 94% |  |  |  |
| 9 Total of 9 in-person visits | 66% |  |  |  |
| 10 Weekly in-person visits during the first four weeks | 71% |  |  |  |
| 11 Weekly contacts (Minimum of 1 in-person visit or telephonic contact each week post-discharge during the active intervention) | 72% |  |  |  |
|  |  |  |  |  |
|  |  |  |  |  |
|  |  |  |  |  |
|  |  |  |  |  |

*Note:* Fidelity Data: Black % = no change; Green %s = improvement from Year 1; Red % = decrease from Year 1. Challenges/Strategies: Black = Coded in Year 1; Blue = New code in Year 2

| **Promoting Continuity**  **Fidelity Results** | | **Qualitative Content Analysis**  **Non-COVID** | **Qualitative Content Analysis**  **COVID** | **Convergent Interpretation** |
| --- | --- | --- | --- | --- |
| **Element** | **%** | **Challenges**   - Complexity of Team-Based Care - Communicating Across Care Systems   **Strategies**   - Optimizing Communication within Members of the Team - Building and Engaging Networks - Trialing Alternative Options - Anticipation of Patient and Caregiver Needs | **Challenges**   - No challenges identified   **Strategies**   - No strategies identified | - Challenges to Promoting Continuity remain consistent from Y1 to Y2 across all sites.   - *Complexity of Team-Based Care - “*Local rehabs and subacute care facilities have been recently bought out or have ongoing staffing challenges, preventing the APRN team from building strong relationships with the facilities.” [Clinical Team Call]   - *Communicating Across Care Systems- “*Patient was readmitted to a non-VA hospital and APRN was unable to reach staff to discuss a plan of care.” [Clinical Team Call] - Most strategies for Promoting Continuity continued to be used from Y1 to Y2 across all sites, with the addition of the TCM emphasis on anticipating patient and caregiver needs.   - *Anticipation of Patient and Caregiver Needs- “*They will also ensure the SNF has the specialty medication for pulmonary hypertension as the UCSF team suggests it can be more complicated to obtain following discharge.” [Clinical Team Call] - Fidelity to Promoting Continuity remains steady from Y1 to Y2, with no COVID-related impact. |
| 12 Patients’ needs, goals, and plans of care communicated within and across care sites | 97% |  |  |  |
| 13 Ensured SNF/Rehab staff had updated plan | 100% |  |  |  |

*Note:* Fidelity Data: Black % = no change; Green %s = improvement from Year 1; Red % = decrease from Year 1. Challenges/Strategies: Black = Coded in Year 1; Blue = New code in Year 2

| **Coordinating Care**  **Fidelity Results** | | **Qualitative Content Analysis**  **Non-COVID** | **Qualitative Content Analysis**  **COVID** | **Convergent Interpretation** |
| --- | --- | --- | --- | --- |
| **Element** | **%** | **Challenges**   - Identifying and Evaluating Best Fit - Limited Resources   **Strategies**   - Building and Engaging Networks - Focus on Plan of Care - Trialing Alternative Options | **Challenges**   - Virtual Visits - Limited Resources - Communicating Across Care Systems   **Strategies**   - Building and Engaging Networks - Anticipation of Patient and Caregiver Needs - Optimizing Communication within Members of the Team | - While Coordinating Care fidelity has improved since Y1, sites continue to experience new site-dependent challenges to fidelity that are both COVID and non-COVID related.   - *Non-Covid: Limited Resources- “*there are long waiting lists for both outpatient palliative and primary care at home teams at may be helpful for possible palliative care involvement.” [Clinical Team Call]   - *COVID: Communicating Across Care Systems- “*APRN was unable to contact attending physician while patient was admitted to the COVID rehab center, as there was a new attending present every day.” [Clinical Team Call] - Fidelity to ensuring community agencies understood patient needs (3.3) and that the services were meeting patients’ needs (3.4) improved from Y1 to Y2 through Building and Engaging Networks, using Strategies to establish appropriate plans care plans, identifying supportive systems, communication strategies and work arounds (Trialing Alt Strategies; Communication Strategies), and anticipating patient and caregiver needs.   - *Trialing Alternative* Options*- “*SITE team coordinated to continue SITE home services that were being delivered prior to rehospitalization.” [Clinical Team Call] - Some sites have explored alternative strategies to address care coordination needs, which may have contributed to improvements in fidelity around community resources.   - *Optimizing Communication within Members of the Team-* “APRN contacted both the patient and the hospitalist daily while inpatient.” [Clinical Team Call] |
| 14 Evaluated health and/ or social service needs | 96% |  |  |  |
| 15 Ensured community agency understood health needs | 79% |  |  |  |
| 16 Assessed if agency resources were meeting patient needs | 96% |  |  |  |
| 17 Priority needs and plans to address them and/or results communicated to the team | 99% |  |  |  |
|  |  |  |  |  |

*Note:* Fidelity Data: Black % = no change; Green %s = improvement from Year 1; Red % = decrease from Year 1. Challenges/Strategies: Black = Coded in Year 1; Blue = New code in Year 2

| **Collaborating with Patients, Caregivers & Team**  **Fidelity Results** | | **Qualitative Content Analysis**  **Non-COVID** | **Qualitative Content Analysis**  **COVID** | **Convergent Interpretation** |
| --- | --- | --- | --- | --- |
| **Element** | **%** | **Challenges**   - Social Determinants of Health - Patient and Caregiver Preferences - Patient and Caregiver Engagement - Complexity of Team-Based Care   **Strategies**   - Establishing Trust - Targeted Patient and Caregiver Engagement - Building and Engaging Networks - Trialing Alternative Options - TCM Care Coordination - Penn Team Collaboration | **Challenges**   - No challenges identified   **Strategies**   - No strategies identified | - In Y2 COVID-related challenges impacting Collaborating with Patients, Caregivers, and Team were no longer present. - Non-COVID challenges remained the same from Y1. - Fidelity data related to care plan implementation and evaluation (4.2, 4.3) improved from Y1 to Y2 with few changes in challenges and strategies. This increase may represent improved documentation and long-term impact of existing strategies.   - *Targeted Patient and Caregiver Engagement Strategies- “*APRN was able to attend PCP visit with a Cantonese-speaking APRN.” [Clinical Team Call]   - *TCM Care Coordination- “*APRN coordinated a call with MD and four of the patient’s family members to discuss patient’s plan of care. APRN contacted MD prior to discussion to provide MD with background information on family challenges around hospice/palliative care.” [Clinical Team Call] |
| 18 Communicated with hospital, health care team and/or community agencies team to get them to work together | 99% |  |  |  |
| 19 Collaborative design, implementation and evaluation of care plans | 93% |  |  |  |
| 20 Care plan updated with providers | 87% |  |  |  |

*Note:* Fidelity Data: Black % = no change; Green %s = improvement from Year 1; Red % = decrease from Year 1. Challenges/Strategies: Black = Coded in Year 1; Blue = New code in Year 2

| **Maintaining Relationships with Patients & Caregivers**  **Fidelity Results** | | **Qualitative Content Analysis**  **Non-COVID** | **Qualitative Content Analysis**  **COVID** | **Convergent Interpretation** |
| --- | --- | --- | --- | --- |
| **Element** | **%** | **Challenges**   - Patient and Caregiver Preferences   **Strategies**   - Establishing Trust - Building and Engaging Networks - Anticipation of Patient and Caregiver Needs | **Challenges**   - Challenges Establishing Trust   **Strategies**   - *Establishing Trust* | - Despite high fidelity scores for Maintaining Relationships with Patients and Caregivers, sites reported new non-COVID challenges and strategies in Y2. While sites are experiencing individual challenges, all continued to experience non-COVID-related challenges due to Patient and Caregiver Preferences.   - *Patient and Caregiver Preferences*- “The primary caregiver works night shifts, so contacting the caregiver has also been difficult.” [Clinical Team Call] - In Y2, more strategies to address non-COVID challenges impacting Maintaining Relationships with Patients and Caregivers were discussed though fidelity remained high.   - *Building and Engaging Networks*- “Social work was contacted to coordinate Meals-on-Wheels, transit, and IHSS services. [Clinical Team Call]   - *Anticipation of Patient and Caregiver Needs*- “APRN should adjust expectations; if a person seems like they might be one to passively distance themselves from the study before the 60-day mark, know that they have to “fast-track” some items to keep the participant engaged. [Clinical Team Call] - No new COVID-related challenges and strategies impacting Maintaining Relationships with Patients and Caregivers were identified. Establishing trust related to COVID continued in Y2 though infrequently discussed and only at VA sites. |
| 21 Documentation of advocacy to achieve goals | 100% |  |  |  |
| 22 Coach patient about preparation for visits and conversations with providers | 99% |  |  |  |
| 23 Advocacy for patients’ goals and preferences with multidisciplinary team | 98% |  |  |  |

*Note:* Fidelity Data: Black % = no change; Green %s = improvement from Year 1; Red % = decrease from Year 1. Challenges/Strategies: Black = Coded in Year 1; Blue = New code in Year 2

| **Engaging Patients & Caregivers** | | | **Qualitative Content Analysis**  **Non-COVID** | **Qualitative Content Analysis**  **COVID** | **Convergent Interpretation** |
| --- | --- | --- | --- | --- | --- |
| **Element** | **%** | **Challenges**   - Need for Goal Alignment - Patient and Caregiver Engagement   **Strategies**   - Focus on Plan of Care - Establishing Trust^3^ - Strategies for Addressing and Setting Goals - Normalization and Validation - Targeted Patient and Caregiver Engagement - Building and Engaging Networks - Trialing Alternative Options - Anticipation of Patient and Caregiver Needs | | **Challenges**   - Caregiver Limitations   **Strategies**   - Trialing Alternative Options | - Fidelity data related to Engaging Patients and Caregivers increased in Y2 from Y1, reflecting the delayed impact of the primarily unchanged challenges and strategies form Y1 into Y2, as well as improved documentation efforts. - Component 6.4 and 6.5 fidelity improvements may be driven by strategies focusing on addressing plan of care and validating APRN experiences.   - *Normalization and Validation-* “VA Team validates the APRN’s frustrations and comments that mental health and substance abuse may complicate this situation.” [Clinical Team Call]   - *Focus on Plan of Care- “*APRN focused on developing a rapport with family and patient prior to initiating ACP discussions.” [Clinical Team Call] |
| 24 Patients’ goals and preferences identified | 97% |  |  |  |  |
| 25 Patients’ goals and preferences updated or reevaluated | 96% |  |  |  |  |
| 26 Caregivers’ goals and preferences identified | 100% |  |  |  |  |
| 27 Caregivers’ goals and preferences updated or reevaluated | **84%** |  |  |  |  |
| 28 Advance planning, care preference discussion, or end of life care conversation | **85%** |  |  |  |  |

*Note:* Fidelity Data: Black % = no change; Green %s = improvement from Year 1; Red % = decrease from Year 1. Challenges/Strategies: Black = Coded in Year 1; Blue = New code in Year 2

| **Managing Symptoms and Other Risks**  **Fidelity Results** | | | **Qualitative Content Analysis**  **Non-COVID** | **Qualitative Content Analysis**  **COVID** | **Convergent Interpretation** |
| --- | --- | --- | --- | --- | --- |
| **Element** | **%** | **Challenges**   - Unmanaged Symptoms - Social Determinants of Health - Patient Medical Complexity - Medication or Equipment Issues - Patient and Caregiver Preferences - Intervention Learning Curve   **Strategies**   - Focus on Plan of Care - Normalization and Validation - Targeted Patient and Caregiver Engagement - Building and Engaging Networks - Trialing Alternative Options - Clarifying or Adapting TCM Processes - TCM Care Coordination | | **Challenges**   - Social Determinants of Health - Patient Medical Complexity - Patient and Caregiver Preferences   **Strategies**   - Building and Engaging Networks - Trialing Alternative Options | - In Y2, medical and social complexity continues to impact fidelity to Managing Symptoms and Other Risks both due to COVID and non-COVID factors. - Most non-COVID challenges were experienced across all sites, and were driven by patient perceptions and behaviors, unmanaged symptoms, and patient medical complexity.   - *Patient Perceptions and Behaviors-* “You have to understand the mentality of a veteran. They don't accept help. They don't want to admit that they are having any problems because they are “warriors,” for lack of another word.” [Leadership Call]   - *Patient Medical Complexity*- “Patient has lung cancer, but his son is not allowing hospice into the home. The VA CLE team believes that because of this reluctance to allow hospice into the home, the patient will end up being readmitted to the hospital.” [Clinical Team Call]   - *Unmanaged Symptoms- “*Patient’s caregiver was unable to manage patient at home due to the 8-10 bowel movements per day and the patient was readmitted to the hospital.” [Clinical Team Call] - Improvements in the Managing Symptoms and Other Risks component may be driven by all sites’ use of strategies aimed at communication and plan of care.   - *Targeted Patient and Caregiver Engagement* *Strategies-* “VA CLE team suggests using motivational interviewing to assist in understanding patient’s perspective.” [Clinical Team Call]   - *Focus on Plan of Care-* “Information on using the inhaler has been posted on patient’s mirror to provide a reminder on usage instructions.” [Clinical Team Call] |
| 29 Patients assessed and addressed for symptom management needs | 99% |  |  |  |  |
| 30 Completion of clinical assessment (check if assessed during contact) | 92% |  |  |  |  |
| 31 Urgent/emergent care plan developed or updated or reinforced | 96% |  |  |  |  |
| 32 Routine symptom management plan updated and reinforced | 100% |  |  |  |  |
|  |  |  |  |  |  |

*Note:* Fidelity Data: Black % = no change; Green %s = improvement from Year 1; Red % = decrease from Year 1. Challenges/Strategies: Black = Coded in Year 1; Blue = New code in Year 2

| **Educating/Promoting Self-Management**  **Fidelity Results** | | | **Qualitative Content Analysis**  **Non-COVID** | **Qualitative Content Analysis**  **COVID** | **Convergent Interpretation** |
| --- | --- | --- | --- | --- | --- |
| **Element** | **%** | **Challenges**   - Patient and Caregiver Engagement^3^   **Strategies**   - Reinforcing Education and Self-Management^3^ | | **Challenges**   - No challenges identified   **Strategies**   - No strategies identified | - There were no COVID related mentions of Education and Promotion of Self-Management challenges and strategies in Clinical Team Calls and those that were discussed were infrequent (only one site). - All fidelity elements related to Education and Promotion of Self-Management increased to 100% in Y2, which may be driven by long-term application of strategies for common challenges.   - *Patient and Caregiver Engagement*- “Patient has a pill box but is not consistent with timing.” [Clinical Team Call]   - *Reinforcing Education and Self-Management-* “APRN provided print copies of medication information to support verbal medication education during first home visit.” [Clinical Team Call] |
| 33 Education about connection between plan of care and achieving goals | 100% |  |  |  |  |
| 34 Education to recognize symptoms | 100% |  |  |  |  |
| 35 Preparation re: symptom management skills | 100% |  |  |  |  |
| 36 Education about how to implement emergency plan* | 100% |  |  |  |  |
| 37 Use of teach-back, coaching, problem solving | 100% |  |  |  |  |
| 38 Educated about self-care management | 100% |  |  |  |  |

*Note:* Fidelity Data: Black % = no change; Green %s = improvement from Year 1; Red % = decrease from Year 1. Challenges/Strategies: Black = Coded in Year 1; Blue = New code in Year 2

**Table S4**

*All Enrolled MIRROR-TCM Study Patient Demographic Characteristics ^a^ (n = 908)*

|  | **TCM Group**  **N (%) or**  **mean ± sd (range)** | **Control Group**  **N (%) or**  **mean ± sd (range)** | **p-value** |
| --- | --- | --- | --- |
| N | 443 | 465 |  |
| Age, *Years* | 77.3 ± 8.1  (65-102) | 76.7 ± 8.2  (65-104) | 0.278 |
| Male | 317 ( 71.6%) | 306 ( 66.1%) | 0.076 |
| Race: White | 287 (64.8%) | 281 (60.4%) | 0.480 |
| Black/African American | 93 (21.0%) | 111 (23.9%) |  |
| Asian | 33 ( 7.4%) | 36 ( 7.7%) |  |
| Other (Multiracial, American Indian, Native Hawaiian/Pacific Islander) | 20 ( 4.5%) | 28 ( 6.0%) |  |
| No Response | 10 ( 2.3%) | 15 ( 1.9%) |  |
| Hispanic/Latino | 21 ( 4.8%) | 20 ( 4.3%) | 0.749 |
| Education |  |  | 0.394 |
| Less than 12 years of school | 57 ( 12.9%) | 59 (12.7%) |  |
| High School Diploma/GED | 121 ( 27.3%) | 139 (29.9%) |  |
| Some College / 2-Year Degree | 166 (37.5%) | 155 (33.3%) |  |
| College Degree or higher | 98 ( 22.1%) | 105 (22.6%) |  |
| No Response | 1 ( 0.2%) | 7 ( 1.5%) |  |
| Marital Status |  |  | 0.592 |
| Single | 51 (11.5%) | 58 (12.5%) |  |
| Married/Partnered | 182 (41.1%) | 179 (38.5%) |  |
| Divorced/Separated | 98 (22.1%) | 99 (21.3%) |  |
| Widowed | 112 (25.3%) | 129 (27.7%) |  |
| Veteran Status | 242 ( 54.6%) | 240 (51.6) | 0.296 |
| Living situation |  |  | 0.761 |
| Lives alone | 157 ( 35.4%) | 169 (36.3%) |  |
| Lives with |  |  |  |
| Spouse | 126 ( 28.4%) | 123 (26.5%) |  |
| Adult Child | 32 ( 7.2%) | 49 (10.5%) |  |
| Other (family, friend, paid caregiver) | 46 (10.4%) | 55 (11.8%) |  |
| Multiple | 72 ( 16.3%) | 64 (13.8%) |  |
| No Response | 10 ( 2.3%) | 5 ( 1.1%) |  |
| Requires Any Activities of Daily Living Assistance | 179 ( 40.4%) | 202 (43.4%) | 0.276 |

*Note:* ^a^ The data for Site D’s enrollments are not presented.

**Figure S1**

*Mean Transitional Care Model Intervention Fidelity Score by Time, Cohort, and Site, by Cohort*

|   Cohort 1  Cohort 3  Cohort 2 | **SITE A**  *All enrolled, regardless of cohort, had an average fidelity score above 90%.* |  |
| --- | --- | --- |
|   Cohort 2  Cohort 3 | **SITE B**  *Cohort 2 (2/2021-1/2022) started below 90% and over the first 12 months of implementation achieved over 90%. Cohort 3 (2/2022-1/2023)had an average fidelity score above 90%. No enrollments in cohort 1.* |  |
|   Months  Cohort 2  Cohort 1  Cohort 3 | **SITE C**  *Cohorts 1 (9/2020-1/2021) and 2 (2/2021-1/2022) remain below 90%, though Cohort 2 begins to approach an average score of 34 over 12 months. The final Cohort 3 reaches 90% fidelity (2/2022-1/2023).* |  |
|  | | |

*Note*: The X-axis represents the month of cohort recruitment. The solid line (1) is Cohort 1, the fine dashed line (2) is Cohort 2, and the wide dashed line (3) is Cohort 3. The Y-axis represents the fidelity score. A score of 34 out of 38 indicates 90% fidelity to delivering the intervention as intended (100%). Cohort 1 (solid line) had a 5-month recruitment period, while Cohorts 2 (dotted line) and 3 (dashed line) had a 12-month recruitment period. Data for the healthcare system that dropped out of the study are not presented. Data for Site D, which dropped out of the study, are not included.
